# Draft Genome Sequence of Bacillus velezensis Strain E68, Isolated from an Oil Battery

**DOI:** 10.1128/MRA.00332-20

**Published:** 2020-06-11

**Authors:** Nathan Liang, Suha Jabaji

**Affiliations:** a Department of Plant Science, McGill University, Montreal, Quebec, Canada; University of Maryland School of Medicine

## Abstract

Bacillus velezensis strain E68 is a biosurfactant-producing bacterium isolated from an oil battery near Chauvin, Alberta, Canada. Strain E68 exhibited antimicrobial activity against fungal pathogens and could potentially serve as a biological control agent. Its genome was sequenced and annotated, revealing the presence of multiple lipopeptide biosynthetic gene clusters.

## ANNOUNCEMENT

Bacillus velezensis is a species of Gram-positive endospore-forming bacteria capable of promoting plant growth and producing biologically active compounds that suppress a broad spectrum of microorganisms (1). Numerous strains of *B. velezensis* exhibit antifungal activity against a range of human- and plant-pathogenic fungi (2). The suppression of microbial growth by *B. velezensis* is partly linked to the production of cyclic lipopeptides (3). Cyclic lipopeptides are biosurfactants which demonstrate antifungal activity, aid in biofilm formation, and can induce systemic resistance in host plants (4). Bacteria isolated from petroleum oil or soil contaminated with it often demonstrate elevated production of biosurfactants, which help solubilize the hydrocarbon carbon sources (5). As a result, they are attractive candidates for use as biological control agents. Bacterial strain E68, previously identified as B. subtilis, was isolated from an oil battery near Chauvin, Alberta, Canada, and was characterized as a top biosurfactant producer (6). Whole-genome sequencing of strain E68 will provide a basis for understanding its biocontrol mechanisms and facilitating its applications in the future.

A single colony of strain E68 was used to inoculate 10 ml of LB broth. It was then incubated at 37°C overnight, with shaking at 140 rpm. Genomic DNA was extracted from strain E68 using a DNeasy blood and tissue kit (Qiagen, Germany) following the protocol for Gram-positive bacteria. Library preparation and genome sequencing were performed at IDseq, Inc. (Sacramento, CA, USA). The DNA was fragmented using a Covaris E220 sonicator. Then, a paired-end sequencing library was prepared using a Nextera XT DNA library preparation kit (Illumina, USA). Genome sequencing was carried out on a HiSeq X instrument, using a 2 × 150-bp protocol, producing 24,785,553 sequencing reads. All software was used with default settings unless specified otherwise. The quality of the reads was assessed with FastQC v.0.11.8 (7). The reads were then quality trimmed and adapters were removed using Trim Galore v.0.6.5 (8). The trimmed reads were assembled *de novo* using SPAdes v.3.13.0 with k-mer sizes of 21, 33, 55, 77, 99, and 127 (9). This resulted in an assembly with 145 contigs. The contigs were ordered and combined into scaffolds using MeDuSa v.1.6 with the –d setting enabled to estimate gap lengths between pairs of contigs (10). Medusa was given the following set of 5 genomes of Bacillus velezensis strains (RefSeq assembly accession number) for scaffolding purposes: CBMB205 (GCF_002117165.1), FZB42 (GCF_000015785.2), JS25R (GCF_000769555.1), OB9 (GCF_001266815.1), and B26 (GCF_001266825.1). Scaffolds under 200 bp or containing contaminants as detected by NCBI’s contamination screen were removed from the assembly. This resulted in a final assembly of 69 scaffolds and a total length of 3,862,910 bp. The assembly featured a GC content of 46.65%, an *N*_50_ value of 3,717,019, and an average sequencing depth of 1,809×. Assembly statistics were provided by QUAST v.5.0.2 (11), and sequencing depth information was provided by BBMap v.37.36 (12). Strain E68 was identified as Bacillus velezensis following ribosomal multilocus sequence typing analysis (https://pubmlst.org/rmlst/) (13). The draft genome was annotated with NCBI’s Prokaryotic Genome Annotation Pipeline v.4.11 (14). Annotation detected 3,637 coding DNA sequences, 111 RNA genes, 12 complete rRNAs, 86 tRNAs, 5 noncoding RNAs (ncRNAs), and 75 pseudogenes. Biosynthetic gene clusters were identified with antiSMASH v.5.1.1 (15). The genome of strain E68 contained gene clusters for the cyclic lipopeptides surfactin, fengycin, and bacillomycin D, as well as the antimicrobials bacillaene, difficidin, macrolactin H, bacillibactin, and bacilysin.

### Data availability.

This whole-genome shotgun project has been deposited at DDBJ/ENA/GenBank under the accession number JAAECG000000000. The version described in this paper is the first version, JAAECG010000000. The sequencing reads have been deposited in the NCBI SRA under accession number PRJNA603554.

## References

[1] Rabbee MF, Ali MS, Choi J, Hwang BS, Jeong SC, Baek K 2019 Bacillus velezensis: a valuable member of bioactive molecules within plant microbiomes. Molecules 24:1046. doi:10.3390/molecules24061046. PMC6470737 30884857

[2] Devi S, Kiesewalter HT, Kovács R, Frisvad JC, Weber T, Larsen TO, Kovács ÁT, Ding L 2019 Depiction of secondary metabolites and antifungal activity of Bacillus velezensis DTU001. Synth Syst Biotechnol 4:142–149. doi:10.1016/j.synbio.2019.08.002. 31508511 PMC6719288

[3] Caulier S, Nannan C, Gillis A, Licciardi F, Bragard C, Mahillon J 2019 Overview of the antimicrobial compounds produced by members of the Bacillus subtilis group. Front Microbiol 10:302. doi:10.3389/fmicb.2019.00302. 30873135 PMC6401651

[4] Ongena M, Jacques P 2008 Bacillus lipopeptides: versatile weapons for plant disease biocontrol. Trends Microbiol 16:115–125. doi:10.1016/j.tim.2007.12.009. 18289856

[5] Das K, Mukherjee AK 2007 Crude petroleum-oil biodegradation efficiency of Bacillus subtilis and Pseudomonas aeruginosa strains isolated from a petroleum-oil contaminated soil from North-East India. Bioresour Technol 98:1339–1345. doi:10.1016/j.biortech.2006.05.032. 16828284

[6] Rani M, Weadge JT, Jabaji S 2020 Isolation and characterization of biosurfactant-producing bacteria from oil well batteries with antimicrobial activities against food-borne and plant pathogens. Front Microbiol 11:64. doi:10.3389/fmicb.2020.00064. 32256455 PMC7093026

[7] Andrews S 2010 FastQC: a quality control tool for high throughput sequence data. https://www.bioinformatics.babraham.ac.uk/projects/fastqc/.

[8] Krueger F 2015 Trim Galore!: a wrapper tool around Cutadapt and FastQC to consistently apply quality and adapter trimming to FastQ files. https://www.bioinformatics.babraham.ac.uk/projects/trim_galore/.

[9] Bankevich A, Nurk S, Antipov D, Gurevich AA, Dvorkin M, Kulikov AS, Lesin VM, Nikolenko SI, Pham S, Prjibelski AD, Pyshkin AV, Sirotkin AV, Vyahhi N, Tesler G, Alekseyev MA, Pevzner PA 2012 SPAdes: a new genome assembly algorithm and its applications to single-cell sequencing. J Comput Biol 19:455–477. doi:10.1089/cmb.2012.0021. 22506599 PMC3342519

[10] Bosi E, Donati B, Galardini M, Brunetti S, Sagot M-F, Lió P, Crescenzi P, Fani R, Fondi M 2015 MeDuSa: a multi-draft based scaffolder. Bioinformatics 31:2443–2451. doi:10.1093/bioinformatics/btv171. 25810435

[11] Mikheenko A, Prjibelski A, Saveliev V, Antipov D, Gurevich A 2018 Versatile genome assembly evaluation with QUAST-LG. Bioinformatics 34:i142–i150. doi:10.1093/bioinformatics/bty266. 29949969 PMC6022658

[12] Bushnell B 2014 BBMap short read aligner, and other bioinformatic tools. https://sourceforge.net/projects/bbmap/.

[13] Jolley KA, Bliss CM, Bennett JS, Bratcher HB, Brehony C, Colles FM, Wimalarathna H, Harrison OB, Sheppard SK, Cody AJ, Maiden MCJ 2012 Ribosomal multilocus sequence typing: universal characterization of bacteria from domain to strain. Microbiology 158:1005–1015. doi:10.1099/mic.0.055459-0. 22282518 PMC3492749

[14] Tatusova T, DiCuccio M, Badretdin A, Chetvernin V, Nawrocki EP, Zaslavsky L, Lomsadze A, Pruitt KD, Borodovsky M, Ostell J 2016 NCBI Prokaryotic Genome Annotation Pipeline. Nucleic Acids Res 44:6614–6624. doi:10.1093/nar/gkw569. 27342282 PMC5001611

[15] Blin K, Shaw S, Steinke K, Villebro R, Ziemert N, Lee SY, Medema MH, Weber T 2019 antiSMASH 5.0: updates to the secondary metabolite genome mining pipeline. Nucleic Acids Res 47:W81–W87. doi:10.1093/nar/gkz310. 31032519 PMC6602434

